# Can Polyhydroxyalkanoates Be Produced Efficiently From Waste Plant and Animal Oils?

**DOI:** 10.3389/fbioe.2020.00169

**Published:** 2020-03-17

**Authors:** Arthy Surendran, Manoj Lakshmanan, Jiun Yee Chee, Azlinah Mohd Sulaiman, Doan Van Thuoc, Kumar Sudesh

**Affiliations:** ^1^School of Biological Sciences, Universiti Sains Malaysia, Penang, Malaysia; ^2^USM-RIKEN International Centre for Aging Science (URICAS), School of Biological Sciences, Universiti Sains Malaysia, Penang, Malaysia; ^3^Faculty of Biology, Hanoi National University of Education, Hanoi, Vietnam

**Keywords:** polyhydroxyalkanoates (PHA), polyoxoester, plant oil, animal oil, metabolic engineering, biocompatible, enzymatic degradation

## Abstract

Polyhydroxyalkanoates (PHAs) are a potential replacement for some petrochemical-based plastics. PHAs are polyesters synthesized and stored by various bacteria and archaea in their cytoplasm as water-insoluble inclusions. PHAs are usually produced when the microbes are cultured with nutrient-limiting concentrations of nitrogen, phosphorus, sulfur, or oxygen and excess carbon sources. Such fermentation conditions have been optimized by industry to reduce the cost of PHAs produced commercially. Industrially, these biodegradable polyesters are derived from microbial fermentation processes utilizing various carbon sources. One of the major constraints in scaling-up PHA production is the cost of the carbon source metabolized by the microorganisms. Hence, cheap and renewable carbon substrates are currently being investigated around the globe. Plant and animal oils have been demonstrated to be excellent carbon sources for high yield production of PHAs. Waste streams from oil mills or the used oils, which are even cheaper, are also used. This approach not only reduces the production cost for PHAs, but also makes a significant contribution toward the reduction of environmental pollution caused by the used oil. Advancements in the genetic and metabolic engineering of bacterial strains have enabled a more efficient utilization of various carbon sources, in achieving high PHA yields with specified monomer compositions. This review discusses recent developments in the biosynthesis and classification of various forms of PHAs produced using crude and waste oils from the oil palm and fish industries. The biodegradability of the PHAs produced from these oils will also be discussed.

## Introduction

Bioplastics have received considerable attention in recent years as consumer preferences throughout the world are shifting toward the use of biodegradable commodities. Bioplastics have numerous advantages over conventional, petrochemical-plastics (petro-plastics) due to their inherent biodegradability, sustainability, and environmentally friendly properties (Sudesh et al., 2011; Heng et al., 2016). These fascinating biopolymers represent a possible eco-friendly alternative to petro-plastics, helping in the preservation of limited fossil fuel resources and also in the reduction of greenhouse gas emissions. This makes them an important innovation for sustainable development (Hassan et al., 2013). Bioplastics are made partly or wholly from biomaterials such as sugar cane, polylactides, polyglycolic acids, polyhydroxyalkanoates (PHAs), aliphatic polyesters, and polysaccharides (Lee, 1996; Divya et al., 2013; Shamsuddin et al., 2017). Reviews on some commercially important biomaterials for bioplastics are available (Sudesh and Iwata, 2008). PHAs are one of the most investigated class of bioplastics expected to replace some of the today's petro-plastics, due to their biodegradable, thermoplastic, and mechanical properties (e.g., versatility, elasticity, flexibility, etc.) (Steinbüchel, 2001; Lee et al., 2008; Nielsen et al., 2017). The PHA Market Research Report stated that the market opportunity for PHA is expected to reach almost USD 98 million by the year 2024 (Market, 2019).

PHAs are a family of biologically synthesized carbon-storage polymers in the form of polyesters, having similar mechanical properties to those of petrochemical-based plastics, with the additional advantage of being completely biodegradable (Mumtaz et al., 2010). These renewable polyesters can be produced by various microorganisms in response to various stress conditions (for example, excess carbon or limited phosphate, nitrogen, sulfur, or oxygen) and provide protection from nutrient starvation and extreme conditions (Prieto et al., 2016). A number of Gram-negative and Gram-positive bacteria (more than 55 genera) have been employed for the production of PHAs (Doi, 1990; Ng and Sudesh, 2016). Among the various types of PHA-producing bacteria, *Cupriavidus necator* (formerly known as *Wautersia eutropha, Ralstonia eutropha, Alcaligenes eutrophus*, or *Hydrogenomonas eutropha*) has gained the most attention due to its ability to grow on various carbon substrate and ability to be cultivated to high cell density (Bhubalan et al., 2010; Devi-Nair et al., 2013).

There are over 155 confirmed unique PHA monomer subunits, which determine the properties and functionalities, as well as the biodegradability, of the PHA polymers (Nielsen et al., 2017). Currently, various copolymers such as poly(3-hydroxybutyrate) [P(3HB)] (Doi, 1990), poly(3-hydroxybutyrate-*co*-3-hyroxyvalerate [P(3HB-*co*-3HV)] (Al-Kaddo et al., 2016), poly(3-hydroxybutyrate-*co*-4-hydroxybutyrate) **[**P(3HB-*co*-4HB)] (Lee et al., 2004; Vigneswari et al., 2009), poly(3-hydroxybutyrate-*co*-3-hydroxyhexanoate) [P(3HB-*co*-3HHx)] (Murugan et al., 2016a), and medium chain length (mcl-PHA) (Klinke et al., 1999; Chen et al., 2014) have been produced.

Despite a significant number of studies on PHA and increasing market demand, the deployment of PHAs into the market is still in its early stages. One of the major restrictions for the wide commercialization and industrialization of PHAs is the high cost of production. One of the major reasons for their high cost is the price of the carbon substrate used in the microbial cultivation process, which can account for 45 to 50% of the total production cost (Bugnicourt et al., 2014; Kourmentza et al., 2015). Thus, there is a need to find cheap and efficient alternative substrates to improve the sustainability and economic feasibility of PHA production.

In response to this issue, much research has been conducted into the possibility of using industrial waste streams for fermentation processes to make PHAs economically favorable (Shahzad et al., 2013; Zakaria et al., 2013; Chanasit et al., 2016). Important examples are the utilization of waste plant oils, molasses from the sugar industry, lignocellulosic materials, oil palm shell, pressed fruit fiber, biodiesel waste, and waste animal oil. The residues coming from agriculture constitute the most abundant biomass available on earth, and are a rich source of starting materials for the production of numerous value-added products in a cost-effective manner (Ballinas-Casarrubias et al., 2016). Among the waste streams identified, there is convincing scientific evidence in support of the higher carbon content in oil-based feedstocks (Akiyama et al., 2003). So far, PHA production using waste plant oils (e.g., palm kernel oil, crude palm oil, palm oil, jatropha oil, sludge palm oil, soybean oil) as inexpensive carbon sources has been taken into consideration owing to its widespread availability and renewability (Lee et al., 2008; Sudesh et al., 2011; Rodrigues et al., 2019; Thinagaran and Sudesh, 2019). Different agriculture sectors generate different types of waste. The yearly global production of the major vegetable oils such as palm oil, canola oil, sunflower oil, and soybean oil is increasing tremendously and at the same time the amount of waste generated by these oils is also increasing, which in turn creates disposal problems (Lligadas et al., 2013; Samarth and Mahanwar, 2015; Phang and Lau, 2017). Palm and soybean oil are the major vegetable oils worldwide with annual production amounting to 73.5 and 56.9 million metric tons, respectively, followed by rapeseed oil and sunflower seed oil, with about 27.9 and 19.5 million metric tons, respectively (Statista, 2019). Approximately 80% of these vegetable oils are used for food, while the rest is used in industrial sectors (Samarth and Mahanwar, 2015). In the case of using vegetables oils for food, the waste generated from waste cooking oil (WCO) is abundant. Europe produces approximately 700,000 to 1,000,000 tons of WCO annually, and it was estimated that 40,000 tons of WCO was produced yearly in Asian countries such as China, Malaysia, Indonesia, Thailand, Hong Kong, and India (Ismail, 2005; Kamaruzaman et al., 2013).

Animal oil is another excellent renewable carbon sources for the production of PHA (Shahzad et al., 2013; Van Thuoc et al., 2019). Animal waste from aquaculture site is rich in valuable oils, minerals, enzymes, and pigments that can be turned into value-added products. Few studies have been conducted to use the waste animal oil as raw materials for the biodiesel and PHA production (Riedel et al., 2015; Kusmiyati and Wulandari, 2016; Samat et al., 2018). However, detailed knowledge of using waste animal oils as an alternative raw material for the production of PHA is still lacking.

In the following sections, the biosynthesis of PHA using waste plant and animal oils will be presented and discussed, as well as the physiochemical aspects of different types of PHA polymers produced. Biodegradability is an important property of PHA. Therefore, the biodegradability of the PHA produced from these substrates will be also discussed.

Overall, the aim of this review is to disseminate the recent findings on the production, application, and biodegradation of PHA by presenting a summary of the contemporary understanding and recent progress in the production of PHA using waste plant and animal oils, and along the way, to draw the attention of researchers and stakeholders to investigate and exploit these renewable resources economically.

## Biosynthesis of PHA

PHAs are classified as linear chain polyesters comprising hydroxy acid (HA) monomers linked by an ester bond. This bond is established when the carboxyl group of a monomer is connected to the hydroxyl group of a neighboring monomer (Philip et al., 2007). Biosynthesis of PHA occurs in diverse microbial species in the environment. This includes various bacterial species, cyanobacteria, and archaebacteria. PHAs are classified into two main groups, namely, short chain length PHAs (scl) and medium chain length PHAs (mcl), depending on the number of carbons in the monomers. Scl-PHAs consist of 3–5 carbon atoms and are synthesized by many microorganisms. The model bacterium used in many PHA studies, *C. necator*, also synthesizes scl-PHAs. The most common type of scl-PHA synthesized is the homopolymer of poly(3-hydroxybutyrate) [P(3HB)], which is composed solely of 3-hydroxybutyrate (3HB) monomers. On the other hand, mcl-PHAs consist of monomers with 6–14 carbon atoms, which are mostly biosynthesized by *Pseudomonas* spp. (Mozejko-Ciesielska and Kiewisz, 2016). Microorganisms are also able to biosynthesize copolymers from single or mixed substrates. PHA copolymers are composed of more than one type of monomer. Microorganisms can convert carbon sources into scl copolymers, for example, poly(3-hydroxybutyrate-*co*-4-hydroxybutyrate) [P(3HB-*co*-4HB)] or poly(3-hydroxybutyrate-*co*-3-hydroxyvalerate [P(3HB-*co*-3HV)], mcl copolymers such as poly(3-hydroxyhexanoate-*co*-3-hydroxyoctanoate) [P(3HHx-*co*-3HO)] (Mozejko-Ciesielska and Kiewisz, 2016), or a combination of both scl and mcl monomers such as poly(3-hydroxybutyrate-*co*-3-hydroxyhexanoate) [P(3HB-*co*-3HHx)]. Scl-mcl-PHAs are of particular interest because of their biophysical properties that resemble some commodity plastics.

PHA synthase is the key enzyme in polymerizing PHA using the (*R*)-3-hydroxyacyl-CoA as a substrate. Depending on the subunits, amino acid sequence, and substrate specificity, this synthase can be classified into four groups (class I–IV). PHA synthases under class I, III, and IV were found to polymerize scl monomers, while class II polymerizes mcl monomers. Class I and class II PHA synthases are made up of a single subunit (PhaC) with molecular weights between 61 and 73 kDa (Qi and Rehm, 2001). PHA synthase from the bacterium *C. necator* falls under class I, while the *Pseudomonas putida* PhaC is categorized under class II. Class III and class IV synthases require two types of subunit to be functional. The subunits are named PhaC and PhaE for class III PHA synthases, and PhaC and PhaR for class IV synthases. PhaC and PhaE of class III synthase have molecular weights of 40.3 and 20–40 kDa, respectively. On the other hand, PhaC and PhaR of class IV synthases have molecular weights of 41.5 and 22 kDa, respectively. Some examples of bacteria that express class III and IV synthases are *Allochromatium vinosum* and *Bacillus megaterium*, respectively. To date, crystal structures have only been determined for the scl-PHA synthases from *C. necator* H16 and *Chromobacterium* sp. USM2 (Wittenborn et al., 2016; Chek et al., 2017; Kim J. et al., 2017; Kim Y.-J. et al., 2017).

### Biosynthesis of PHAs Using Waste Plant Oils

The section above described in general that biosynthesis of PHAs is often carried out using sugars, plant oils, and fatty acids. However, usage of these expensive carbon sources are not feasible for scaling up PHA production as it may add a significant cost burden to production. With the current growing interest in utilizing PHA as an alternative plastic material to replace some petro-plastics, it is crucial to source cheaper carbon substrates that can markedly decrease the production cost. Waste plant oils, be it from the edible oil industry or other agricultural sectors, stand as inexpensive and excellent carbon substrates to substitute the commonly used carbon sources in PHA biosynthesis. WCO, for example, is one of the major wastes produced from the human food processing industry. It is estimated that over 29 million tons of WCO is produced annually around the globe (Maddikeri et al., 2012), giving it high potential as an inexpensive carbon substrate for PHA production. The high content of free fatty acids (FFA) in WCO gives it an added advantage to be used as a carbon source for PHA biosynthesis. Song et al. have reported the utilization of waste vegetable oil in the synthesis of various types of mcl-PHAs (C6-16) from *Pseudomonas* sp. strain DR2 (Song et al., 2008). Rao et al. have successfully biosynthesized a P(3HB-*co*-4HB) copolymer using spent palm oil after frying as the main carbon source while co-supplementing with 1,4-butanediol as a precursor compound (Rao et al., 2010). Another study has reported the usage of waste frying oil (rapeseed oil) for the production of P(3HB) in *C. necator* strain H16 (Verlinden et al., 2011). Another team of researchers have also explored the utilization of waste rapeseed oil obtained after frying for PHA biosynthesis via *Pseudomonas* sp. strains G101 and G106 (Mozejko et al., 2011). This study reported the production of mcl-PHAs with *Pseudomonas* sp. strain G101 producing predominantly 3-hydroxyoctanoic acid (3HO) and 3-hydroxydecanoic acid (3HD) monomers, while strain G106 produced significant amounts of 3-hydroxyhexanoate (3HHx) monomers (Mozejko et al., 2011). In a later study by the same group, the production of mcl-PHAs by *Pseudomonas* sp. strain G101 was markedly improved using a pulse-feeding method of waste rapeseed oil, whereby 44% of the total cell biomass was found to contain mcl-PHAs after 41 h of fermentation (Mozejko and Ciesielski, 2014). In a separate study by Mozejko and Ciesielski, saponified waste palm oil (SWPO) was used as the sole carbon source for the production of mcl-PHA using *Pseudomonas* sp. strain G101. It was found that under fed-batch fermentation conditions with a total SWPO fed at 15 g/L, the bacterial strain in the study could produce up to 43% of mcl-PHA by dry cell weight (Mozejko and Ciesielski, 2013).

Kamilah and co-workers have also shown that wild type and engineered strains of *C. necator* were able to utilize WCO (palm oil) in the production of P(3HB) and P(3HB-*co*-3HHx), respectively (Kamilah et al., 2013, 2018). In a separate study, it was reported that *P. putida* strain KT2440 was able to produce high contents of mcl-PHAs when grown via high cell density fermentation using hydrolyzed WCO as the sole carbon source (Ruiz et al., 2019). Kourmentza et al., on the other hand, have reported co-production of P(3HB) polymers along with rhamnolipids when *Burkholderia thailandensis* was employed for the bioconversion of used cooking oil. It was reported that up to 60% of the cell biomass consisted of P(3HB) while 2.2 g/L of rhamnolipids were produced simultaneously (Kourmentza et al., 2018).

Besides WCO, there are other categories of plant oils that are of no value for human consumption that may be used for PHA production. One example is the use of jatropha oil. This oil is extracted from the seeds of the plant *Jatropha curcas* L, which has no food value. It has been shown that by utilizing jatropha oil, *C. necator* strain H16 was able to successfully synthesize P(3HB) homopolymers, which shared similar physicochemical attributes with homopolymers produced via palm oil or other carbon sources. While jatropha oil is known for its toxicity to humans and animals, it did not show any inhibition to the growth of *C. necator* during PHA biosynthesis (Ng et al., 2010). In another study, it was shown that *Pseudomonas oleovorans* (ATCC 29347) was able to assimilate saponified jatropha oil as the sole carbon source to produce the copolymer P(3HB-*co*-3HV) (Allen et al., 2010). In a more recent report, Zainab et al. have explored a series of underutilized inedible oils obtained from Africa and some parts of Asia as sole carbon substrate for PHA biosynthesis. These oils include African elemi oil, bitter apple oil, desert date oil, and *Amygdalus pedunculata* oil (Zainab-L et al., 2018). It was found that wild type and engineered *C. necator* strains were able to metabolize these oils into P(3HB) homopolymer and P(3HB-*co*-3HHx) copolymer, respectively, with these two polymers having high molecular weights (*M*_w_) (500,000–2,400,000 Da). The 3HHx proportions in the copolymers were reported to be as high as 31 mol% when biosynthesized using these oils (Zainab-L et al., 2018).

In the production of palm oil in the palm oil mills in Malaysia, Indonesia, and Thailand, a wet milling method is used, whereby the fresh fruit bunch (FFB) is subjected to various stages of treatment before oil is pressed, purified, and refined. These processes require the usage of large volumes of water. It is estimated that for every ton of FFB, 1.5 m^3^ of water is used. Half of this water ends up as palm oil mill effluent (POME) and is collected in a pond outside of the mill (Mumtaz et al., 2010). During the initial stage of POME discharge, the floating residual oil that is collected is known as sludge palm oil (SPO), which exists as a dark brown solid at room temperature (25°C) and exudes a strong odor. This low-grade oil that is rich in FFA is usually used to make cheap laundry soaps, candles, and animal feed additives. SPO has also been explored as a carbon feedstock for the production of PHA due to the high content of FFAs. In a report published by Kang et al., it was observed that SPO could be utilized by several *Pseudomonas* species to produce a myriad of mcl-PHAs. Among all the strains tested, *P. putida* strain S12 was found to produce the highest yield of elastomeric mcl-PHAs (Kang et al., 2017). A recent study by Thinagaran and Sudesh has revealed that emulsified SPO, when supplemented at 10 g/L to the engineered *C. necator* strain Re2058/pCB113, produced as high as 9.7 g/L of dried cells mass in which 74 wt% contained P(3HB-*co*-21 mol% 3HHx). Further improvements in the yield were achieved by employing a fed-batch fermentation strategy, which resulted in biomass productivity of 1.9 g/L/h and PHA productivity of 1.1 g/L/h (Thinagaran and Sudesh, 2019). Table 1 summarizes all the bacterial strains and the respective waste plant oil feedstocks that were used for PHA production.

**Table 1:** Summary of bacterial strains along with the waste oil carbon feedstocks that are utilized to produce various types of PHAs.

**Bacterial strain**	**Waste carbon feedstock**	**Type of PHA produced**	**Reference**
**PLANT OILS**			
*Pseudomonas* sp. DR2	Waste vegetable oil	mcl-PHAs (C6-C16)	Song et al., 2008
*Pseudomonas* sp. G101 and G106	Waste rapeseed oil	mcl-PHAs	Mozejko et al., 2011
*Pseudomonas* sp. G101	Saponified waste palm oil	mcl-PHAs	Mozejko and Ciesielski, 2013
*P. putida* KT2440	Hydrolyzed waste cooking oil	mcl-PHAs	Ruiz et al., 2019
*P. putida* S12	Sludge palm oil	Elastomeric mcl-PHAs	Kang et al., 2017
*P. oleovorans* ATCC 29347	Saponified jatropha oil	P(3HB-*co*-3HV)	Allen et al., 2010
*C. necator* H16	Spent palm oil + 1,4-butanediol	P(3HB-*co*-4HB)	Rao et al., 2010
*C. necator* H16	Waste frying oil (rapeseed)	P(3HB)	Verlinden et al., 2011
*C. necator* H16	Waste cooking oil (palm oil)	P(3HB)	Kamilah et al., 2013
*C. necator* Re2058/pCB113	Waste cooking oil (palm oil)	P(3HB-*co*-3HHx)	Kamilah et al., 2018
*C. necator* H16	Jatropha oil	P(3HB)	Ng et al., 2010
*C. necator* H16	African elemi oil, bitter apple oil, desert date oil, and *Amygdalus pedunculata* oil	P(3HB)	Zainab-L et al., 2018
*C. necator* Re2058/pCB113	African elemi oil, bitter apple oil, desert date oil, and *Amygdalus pedunculata* oil	P(3HB-*co*-3HHx)	Zainab-L et al., 2018
*C. necator* Re2058/pCB113	Sludge palm oil	P(3HB-*co*-3HHx)	Thinagaran and Sudesh, 2019
*B. thailandensis*	Used cooking oil	P(3HB), rhamnolipids	Kourmentza et al., 2018
**ANIMAL OILS**			
*C. necator* Re2058/pCB113	Low-quality waste animal fat	P(3HB-*co*-3HHx)	Riedel et al., 2015
*C. necator* H16	Low-quality waste animal fat	P(3HB)	Riedel et al., 2015
*C. necator* H16	Tallow	P(3HB-*co*-3HV)	Taniguchi et al., 2003
*C. necator* H16	Emulsified waste fish oil	P(3HB)	Kaesavan, 2014
*C. necator* H16	Emulsified waste fish oil with γ-butyrolactone	P(3HB-*co*-4HB)	Kaesavan, 2014
*C. necator* H16	Emulsified waste fish oil with sodium valerate	P(3HB-*co*-3HV)	Kaesavan, 2014
*P. oleovorans* NRRL B-14683	Crude Pollock oil	mcl-PHA	Ashby and Solaiman, 2008
*P. resinovorans* NRRL B-2649	Crude Pollock oil	mcl-PHA	Ashby and Solaiman, 2008
*P. corrugata* 388	Crude Pollock oil	mcl-PHA	Ashby and Solaiman, 2008
*P. putida* KT2442	Crude Pollock oil	mcl-PHA	Ashby and Solaiman, 2008
*P. oleovorans* NRRL B-778	Crude Pollock oil	mcl-PHA	Ashby and Solaiman, 2008
*P. oleovorans* NRRL B-14682	Crude Pollock oil	mcl-PHA	Ashby and Solaiman, 2008
*Salinivibrio* sp. M318	Waste fish oil and glycerol	P(3HB)	Van Thuoc et al., 2019

### Biosynthesis of PHAs Using Waste Animal Oils

#### Animal Fats

Animal fats are rendered tissue fats that can be obtained from the meat packing industry as by-products. A difficulty with the use of animal fats is that they can be difficult to disperse in water due to their physical characteristics. Lard has a melting range between 32 and 43°C, while for tallow, it is higher than 40°C (Ashby et al., 2001; Patterson, 2011; Sharma et al., 2013). Owing to this phenomenon, some animal fats remain solid throughout the fermentation process, and so the bacteria face difficulty in breaking them down for growth and polymer synthesis (Ashby et al., 2001).

Reuse of waste animal fats as renewable resources for PHA production have nevertheless been reported (Ashby and Foglia, 1998; Taniguchi et al., 2003; Ashby and Solaiman, 2008; Riedel et al., 2012, 2015; Muhr et al., 2013). Approximately 45 g/L of CDW with 60 wt% (w/w) P(3HB-*co*-19 mol% 3HHx) content can be attained by the recombinant *C. necator* Re2058/pCB113 using low-quality waste animal fat (Riedel et al., 2015). Feeding of tallow to *C. necator* H16 could accumulate trace amounts of 3HV (1%) in 80 wt% of PHA content and 7.3 g/L of DCW (Taniguchi et al., 2003). This is contradictory to the fact that by applying the same bacterial strain as well as the same carbon source, only pure P(3HB) could be obtained by Riedel et al. (2015). A recombinant strain *C. necator* Re2058/pCB113, which was constructed to produce high fraction of 3-hydroxyhexanoate (3HHx), has successfully synthesized 49–72 wt% of P(3HB-*co*-3HHx) that consisted of 16–27 mol% of 3HHx composition under various waste animal fats (Riedel et al., 2015).

#### Waste Fish Oil

The fishery industries generate huge quantities of by-products. The normal practice for handling these by-products is to dump them back into the sea. Recently, the European Union Fisheries Commission has approved a revised common fisheries policy to develop a strategy allowing the treatment or upgrading of fish industry by-products to other value-added products (García-Moreno and Pérez-Gálvez, 2017). Pet foods constitute a relatively large marketplace for fishery processing wastes, especially canned pet food containing fishery by-products, which provide polyunsaturated fish oil (Hardy, 1992).

An attempt to use emulsified waste fish oil for the production of PHA with the aid of gum arabic as emulsifier was carried out (Kaesavan, 2014). According to Budde et al. (2011), gum arabic neither influences bacterial growth nor is used as a nutrient source by bacteria. Hence, 2.5 g/L of gum arabic was added into waste fish oil and the mixture was found to be an effective carbon source for the synthesis of PHA by *C. necator* H16. Total cell biomass of 4.85 g/L and P(3HB) content of approximately 73 wt% were reached by using 15 g/L of waste fish oil. Co-feeding of structurally related precursors has also successfully yielded copolymers containing 36 mol% 4HB monomer from γ-butyrolactone and 63 mol% 3HV monomer from sodium valerate, respectively (Kaesavan, 2014).

*Pseudomonas* spp. are well-known for their capabilities to hydrolyze triglycerides using lipase to synthesize mcl-PHA. *P. oleovorans* NRRL B-14683, *P. resinovorans* NRRL B-2649, *P. corrugata* 388, and *P. putida* KT2442 synthesized mcl-PHA from crude Pollock oil (a by-product of the Alaskan fishing industry) ranging from 6 to 53% PHA content. All the mcl-PHA polymers produced were primarily composed of the monomers 3-hydroxyoctanoate (3HO) and 3-hydroxydecanoate (3HD) amounting to 75% or higher of the total monomers present. However, come uncommon strains of *P. oleovorans* could only synthesize P(3HB) homopolymer from the same carbon feedstock, such as *P. oleovorans* NRRL B-778 and *P. oleovorans* NRRL B-14682 (Ashby and Solaiman, 2008).

*Salinivibrio* sp. M318, a halophilic bacterium isolated from fermenting shrimp paste, was recently reported to be capable of producing P(3HB) using mixtures of waste fish oil and glycerol as sources of carbon together with fish sauce as a nitrogen source (Van Thuoc et al., 2019). A production yield of up to 10 g/L of DCW and 51.7 wt% of P(3HB) were achieved during growth phase after 48 h of shake flask batch cultivation, and reached up to 69.1 g/L of DCW and 51.5 wt% of P(3HB) content after 78 h of fed-batch cultivation. The ability to synthesize the copolymers P(3HB-*co*-5 mol% 4HB) and P(3HB-*co*-24 mol% 3HV) was also discovered by supplying structurally related precursors to the culture medium. If the by-products from the fisheries industry could be harnessed more widely as carbon feedstocks, there certainly appears to be great potential for their use for bacterial PHA production at low cost. The summary of all the bacterial strains along with the waste animal oils that are used for PHA production is reflected in Table 1.

### Physicochemical Properties of Some Common PHAs

#### P(3HB)

P(3HB) is a stiff material (Doi, 1990) with a high Young's modulus value of 3.5 GPa and a high tensile strength of 43 MPa. The elongation to break of P(3HB) is 5, in comparison to that of polypropylene (PP) which is around 400%. Thus, P(3HB) possesses low ductility. The melting temperature (*T*_m_) of P(3HB) is about 180°C, which is near to its thermal degradation temperature (185°C). This makes P(3HB) unsuitable for thermal processing, especially at higher temperatures. Furthermore, a high glass transition temperature (*T*_g_) of 4°C shows that the P(3HB) homopolymer is highly crystalline when compared to low-density polyethylene (LDPE) or PP (Doi, 1990). The average molecular weight (*M*_w_) of P(3HB) is in the range of 1 × 10^4^ to 3 × 10^6^ Da with a polydispersity of ~2 (Doi, 1990).

From a biotechnological point of view, an *Escherichia coli* XL1-Blue/pSYL105 transformant harboring the PHA synthase gene (*pha*C) of *C. necator* could synthesize ultra-high-molecular weight P(3HB) homopolymer with from 3 × 10^6^ to 11 × 10^6^ Da. The elongation to break of this polymer was 58%, the Young's modulus was 1.1 GPa, and the tensile strength was 62 MPa, demonstrating some improvements in strength and flexibility compared to standard P(3HB) (Kusaka et al., 1998, 1999).

Recently, *Azotobacter vinelandii* OPNA has also been reported to be capable of producing ultra-high-molecular weight P(3HB), reaching 3 × 10^6^ to 11 × 10^6^ Da. This polymer exhibited improved tensile strength and elongation to break without affecting its original biodegradability (Peña et al., 2014).

#### P(3HB-*co*-3HV)

By introducing a second monomer into the PHA backbone, the ductility of P(3HB) can be enhanced as the crystalline phase is reduced (Doi et al., 1995; Yu, 2007). The presence of other monomers can make a significant contribution to improving the mechanical properties compared to the homopolymer (Du et al., 2001). The monomer 3-hydroxyvalerate (3HV) can be synthesized from structurally related carbon sources such as sodium valerate and sodium propionate. The *T*_m_ of P(3HB-*co*-2 mol% 3HV) is 165°C (Eggink et al., 1992), that of P(3HB-*co*-25 mol% 3HV) is 137°C, and that of P(3HB-*co*-70 mol% 3HV) is 87°C, which is far lower than the P(3HB) homopolymer (Holmes, 1988; Singh et al., 2015). The *T*_g_ for P(3HB) homopolymer ranges from 2.5 to 10°C, and this can be decreased to −16°C with 90 mol% 3HV composition (Singh et al., 2015).

The crystallinity of P(3HB-*co*-3HV) is maintained at 60% or higher regardless of the fraction of 3HV monomer present in copolymer chain (Holmes, 1988). The high crystallinity of P(3HB-*co*-3HV) is due to the isodimorphism behavior of this copolymer, whereby the transformation of crystallization from the P(3HB) lattice to the P(3HV) lattice occurs at around 37–40 mol% 3HV (Kunioka et al., 1989; Liu et al., 2014; Wang et al., 2016). At this fraction of 3HV, both the 3HB and 3HV monomers, which are similar in size, can co-crystallize into the same lattice (Doi, 1990; Grigore et al., 2019). This phenomenon only takes place in copolymers of P(3HB-*co*-3HV).

#### P(3HB-*co*-4HB)

P(3HB-*co*-4HB) and P(4HB) are other candidates with commercial value due to their desired properties in medical tissue engineering as absorbents and in the pharmaceutical field as controlled drug delivery agents. Addition of 4HB monomer into P(3HB) polymer results in a copolymer that is hydrolyzable not only by PHA depolymerases but also lipases and esterases (Mukai et al., 1993; Ch'ng and Sudesh, 2013). This is because the chemical structure of 4HB has no alkyl side chains attached to the PHA backbone (Mukai et al., 1993; Saito et al., 1996).

The elongation to break of P(3HB-*co*-4HB) ranges from 5 to 1,000% as the 4HB composition increases from 0 to 100 mol% (Singh et al., 2015). The copolymer exhibits properties from a hard and crystalline plastic to very elastic rubbers (Saito et al., 1996). The characteristics of P(4HB) homopolymer is very similar to ultra-high-molecular-weight polyethylene. When the 4HB fraction was increased from 64 to 100 mol%, it could be considered as a strong elastic polymer with the ability to extend with force and then return to its original position since the tensile strength ranged from 17 to 104 MPa, unlike P(3HB): up to 2.5 times the strength of P(3HB) (Saito et al., 1996; Bhubalan et al., 2010; Grigore et al., 2019).

### Copolymers of 3HB With Medium Chain Length Monomers

In most cases, scl-PHA such as P(3HB) is highly crystalline with poor tensile strength while mcl-PHA tends to be amorphous and very elastomeric. The former is too brittle whereas the latter can be too sticky to be processed conveniently. However, a mcl-PHA, P(2.1 mol% 3HD-*co*-97.9 mol% 3HDD), produced by *Pseudomonas entomophila* LAC25 containing almost pure 3-hydroxydodecanoate (3HDD) and a small fraction of 3HD, has the widest difference between *T*_g_ (−49.3°C) and *T*_m_ (82.4°C) (Chung et al., 2011). High contents of long-chain monomers favor side-chain crystallization, unlike the amorphous conventional mcl-PHA, which generally have a high composition of 3HO and 3HD (Ouyang et al., 2007; Ma et al., 2009; Chung et al., 2011). Due to this unusual effect, Chung et al. (2011) proved that the polymer produced by *P. entomophila* LAC25 consisting of almost pure 3HDD could lead to improved mcl-PHA properties.

Alternatively, incorporation of a minor quantity of 3HHx (5 mol%) into P(3HB) can reduce the melting point to below 155°C (Matsusaki et al., 2000; Loo et al., 2005). In fact, Grigore et al. (2019) revealed that P(3HB-*co*-5.9 mol% 3HHx) had the highest elongation to break value of 163%. When discussing about the molecular weight of P(3HB-*co*-3HHx), Doi (1990) and Riedel et al. (2012) revealed that a decrement in molecular weight (2–11 × 10^5^ Da) was observed when the 3HHx molar fraction became higher. Unexpectedly, a slight increase in the molecular weight was discovered by Murugan et al. (2017) using polymers with 4–15 mol% 3HHx in their studies. The molecular weight was between 5.47 × 10^5^ and 6.85 × 10^5^ Da and the viscoelastic behavior further supported their findings using a rheometer (Murugan et al., 2017). Therefore, developing combinations of scl-mcl monomers can produce a polymer that overcomes the defects of each monomer to possess soft and flexible properties.

On the other hand, when the 3HHx composition increased from 0 to 17 mol%, the tensile strength decreased from 43 to 20 MPa while the elongation to break improved from 6 to 850% (Doi et al., 1995). The degree of X-ray crystallinity of solvent-cast copolymer films decreased from 60 to 18% as the 3HHx fraction increased from 0 to 25 mol% (Doi et al., 1995). This finding was also supported by Volova et al. (2016), who used X-ray structure analysis to demonstrate a significant reduction in the crystalline to amorphous ratio of different P(3HB-*co*-3HHx) samples by increasing the 3HHx monomer fraction. This phenomenon suggests that 3HHx monomers are excluded from the P(3HB) crystalline phase and therefore reduce the overall crystallinity (Doi et al., 1995). Yu (2007) reported that the crystal lattice of P(3HB-*co*-3HHx) is reduced to about half that of the P(3HB) lattice. The *T*_m_ also decreased steeply from 180 to 52°C with similar 3HHx molar fractions (0 to 25 mol%) and *T*_g_ was reduced from 4 to −4°C (Doi et al., 1995). Interestingly, with 3HHx fractions >60 mol%, the elongation was almost 40–50 times higher than with 12 mol% 3HHx monomer. The Young's modulus also decreases with greater proportions of 3HHx, from 1,286.4 (12 mol% of 3HHx) to 217.0 MPa (68 mol% of 3HHx) (Volova et al., 2016).

### Factors Affecting the Efficient Utilization of Waste Plant and Animal Oils for PHA Production

**Figure 1:**
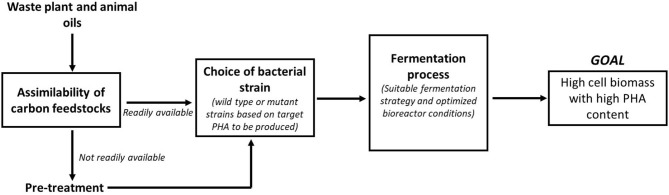
Factors affecting the efficient PHA production using waste plant and animal oils. All the factors outlined are dependent on each other at every level before and during the fermentation process in order to achieve the desired goal of high cell biomass with high PHA content.

Waste oils are indeed an attractive carbon feedstock for PHA production due to its inexpensive cost and availability. However, the challenge remains on how efficiently these waste feedstocks can be used by the bacterial strains for PHA production. One of the major factors that determine efficient utilization of these waste oils is its assimilability by the microbes for PHA biosynthesis. Although feedstocks such as SPO are rich in FFAs, it was found to solidify instantaneously when added to the culture medium (Thinagaran and Sudesh, 2019) causing the oil to be not available homogenously in the bacterial culture. This has resulted in the conversion yield of SPO to biomass to be lower than conversion yield of CPKO to biomass (Thinagaran and Sudesh, 2019). SPO was made readily available for the bacterial strains when emulsified using surfactants. It also ensured that the oil droplets were dispersed more homogenously in the culture medium resulting in improved cell biomass compared to the non-emulsified SPO. Similar strategies were also employed by Riedel et al. when biosynthesis of PHA was performed using waste animal fats and tallow, which exist as solid in room temperature (Riedel et al., 2015). Another important factor in determining efficient utilization of waste oils is the choice of bacterial strain for PHA biosynthesis. While many *Pseudomonas* strains are able to efficiently utilize the waste oils to produce mcl PHAs as shown in the previous section, strains such as *C. necator*, which is also the model PHA producing strain is able to utilize oils to produce only scl-PHA [P(3HB)] in its wild-type form. This homopolymer is less favorable for industrial applications due to its brittle nature. As such, the wild-type *C. necator* has been extensively engineered by various genetic tools to enable the biosynthesis of scl-mcl PHA [e.g., P(3HB-*co*-3HHx)] using waste plant and animal oils. The scl-mcl PHAs are more durable for industrial applications due to their softness and high flexural strength. Besides that, the PHA production efficiency using waste oil feedstocks can be enhanced by altering the fermentation strategy that is involved in the PHA biosynthesis. More often, the biosynthesis of PHA are conducted as batch fermentations in shake flasks to determine the ability of bacterial strains to utilize certain carbon feedstocks for PHA production. Based on the observations in shake flask experiments, the biosynthesis is usually scaled up in a bioreactor system and this is where many parameters have to be carefully studied and manipulated to generate a maximum conversion yield of carbon feedstock to biomass. Based on the reported studies using bioreactors for PHA production, it was found that fed-batch fermentation strategy and/or pulse-feeding of carbon feedstocks are the preferred methods for efficient PHA production using waste plant or animal oils (Mozejko and Ciesielski, 2013, 2014; Riedel et al., 2015; Zainab-L et al., 2018; Thinagaran and Sudesh, 2019). Employing a suitable fermentation strategy also goes hand in hand with a proper control of some of the key bioreactor conditions such as dissolved oxygen (DO), impeller design, agitation speed of impeller, aeration rate, pH, and temperature control for an efficient utilization of waste oil feedstocks for PHA production. While foaming indicates a good bacterial growth during fermentation process, it may pose a serious problem in smaller scale bioreactors if not handled properly. The bacterial cultures may flow out continuously causing dilution of the fermentation medium and inefficient utilization of carbon feedstocks. Proper control of foaming is necessary either by using a suitable anti-foam compound or a mechanical foam breaker. This is especially important when high cell density fermentations are performed. All these factors are interrelated for an efficient PHA production process and therefore cannot be omitted. Figure 1 shows the involvement of these factors in the entire PHA biosynthesis process. Answering the question posed in the title of this review, “Can polyhydroxyalkanoates be produced efficiently from waste plant and animal oil?,” the answer is a yes. These waste plant and animal oil feedstocks can be definitely used for an efficient PHA production, providing the three major factors proposed in this section are properly optimized. In fact, waste plant and animal oils are a potential good source of carbon feedstocks for an economical and sustainable PHA production commercially.

### Downstream Processing of PHA

After fermentation, the downstream processing of PHA plays a noteworthy role in production cost. Unlike upstream processing, reports on the downstream processing and purification of PHAs are scarce. PHAs are present as amorphous solid granules suspended in the cytoplasm of the cells with 5–10 wt% of water along with a protein component containing PHA synthase and PHA depolymerase that binds strongly to the PHA granule (McCool et al., 1996; Bresan et al., 2016). The separation of PHA from non-PHA cell mass (NPCM) is technically challenging because both PHA and NPCM are in the solid phase. Some downstream processing strategies are discussed in this section (Figure 1).

### Chemical Treatment

To date, various methods have been established to separate and purify PHAs from NPCM with varying efficiencies. More often than not, the PHA is purified by either solubilizing the PHA or NPCM dissolution. Chemical treatments can be broadly divided into two categories. One is solvent extraction, where PHA molecules are dissolved in appropriate solvents and later the PHA is precipitated. The second method is digestion. While the solvent extraction techniques involve the solubilization of PHA granules, in the digestion technique, NPCM is dissolved.

The most commonly used solvents are chlorinated hydrocarbons, namely, chloroform, 1,2-dichloroethane, methyl ethyl ketone (MEK), and methyl isobutyl ketone (MIBK), or some cyclic carbonates such as ethylene carbonate and 1,2-propylene carbonate (Lafferty and Heinzle, 1978; Ramsay et al., 1994; Jiang et al., 2006). PHA is precipitated using non-solvents like methanol or ethanol (Kunasundari and Sudesh, 2011). The solvent extraction method is considered to be the most effective in terms of purity. Optimized PHA extraction in *C. necator* with chloroform was able to recover 94% of the polymer with 98% purity (Fiorese et al., 2009). This method is commonly used on the laboratory scale but has not been widely adopted on the pilot scale and for commercial processing. This is due to its high operational and capital cost along with waste disposal issues (Tamer et al., 1998; Yu and Chen, 2006; McChalicher et al., 2010).

The digestion method can be further sub-classified into chemical digestion and enzymatic digestion. In chemical digestion, the NPCM is digested by sodium hypochloride or surfactants like sodium dodecyl sulfate (SDS), Triton X-100, palmitoyl carnitine, or betaine. Sodium hypochloride has a strong non-selective oxidizing property that can digest the NPCM (Yu and Chen, 2006). Sequential treatment with sodium hypochloride and a mixture of surfactants promoted the recovery of PHA with 50% reduced cost when compared to solvent extraction (Yu, 2009; Divyashree and Shamala, 2010). Even though chemical digestion has a low operating cost, it is considered unattractive due to complications in wastewater treatment and also the cost involved in the utilization of surfactants (Kunasundari and Sudesh, 2011).

The recovery of PHA via enzymatic degradation is a complex procedure. In general, the dissolution of NPCM is followed by heat treatment, enzymatic hydrolysis, and surfactant washing. Various types of enzymes have been studied for their activity to digest NPCM, but the most commonly employed is protease. The enzymatic degradation technique is highly efficient and yields up to 92% purity because the enzymes are specific in their action. However, the cost of enzymes and the complexity of the procedure make it unattractive for industry (Middelberg, 1995; Kapritchkoff et al., 2006).

### Mechanical Treatment

Mechanical disruption is the most common method for the removal of intracellular protein. Bead milling and high-pressure homogenization are the most commonly used mechanical disruption techniques for PHA recovery. Mechanical disruption is the most favored method for PHA recovery due to the minimal damage caused to the polymer during the recovery process (Kunasundari and Sudesh, 2011). Additionally, mechanical disruption is cost-effective and environmentally friendly.

#### Bead Mill

The basis of a bead mill is the transfer of shear force from the beads to the cell membrane. The key parameters governing the efficiency of cell disruption are number of beads, bead size, residence time distribution (RTD), and agitation speed (Middelberg, 1995; Tamer et al., 1998). Bead mill disruption is highly favored for PHA recovery as it consumes minimal electrical power and is not susceptible to blockage. No significant interrelationship was observed between the diameter of the bead and cell disruption. However, the smaller size beads cause micronization of PHA (Tamer et al., 1998). The major limitations of this process are the long duration and the large number of parameters to be optimized (Kunasundari and Sudesh, 2011).

#### High-Pressure Homogenization

In high-pressure homogenization, the cell membrane is disrupted under high pressure through a restricted orifice discharge. The parameters to be considered are temperature, pressure, number of passes, and the design of the orifice (Geciova et al., 2002). High-pressure homogenization was found to be less efficient than bead milling when P(3HB) was recovered from *Azohydromonas latus* cells (Kelly and Muske, 2004). Apart from the mechanical parameters, the cell wall of the bacteria plays a central role in the PHA recovery. For instance, Gram-negative bacteria are more difficult to disrupt than the Gram-positive due to the presence of a lipid layer on the surface of the Gram-negative cells. The high pressure in the homogenization process can also cause a thermal degradation of the PHA (Diels and Michiels, 2006).

### Biological Recovery

**Figure 2:**
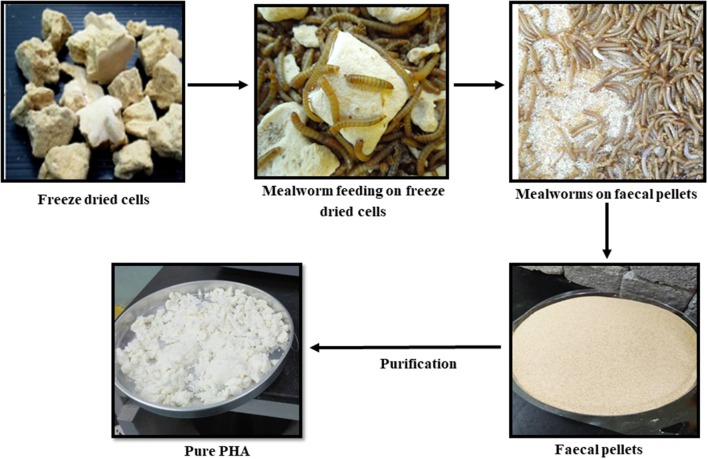
Biological recovery process of PHA from bacterial cells by using mealworms.

Biological recovery is a novel method that bypasses most of the complicated processes mentioned above. The concept is based on utilizing animals, which are fed with dried bacterial cells containing PHA. The animals will digest the NPCM and defecate the PHA (Murugan et al., 2016b; Kunasundari et al., 2017). Mealworms and rats are attractive animal models to be used for PHA purification (Murugan et al., 2016b; Ong et al., 2018a). A detailed study was conducted to evaluate the efficiency of PHA recovery from *C. necator* cells by mealworms as well as the purity of the recovered PHA (Murugan et al., 2016b). The purity of PHA recovered from mealworms was lower than that recovered by rats (Ong et al., 2018b). However, washing the fecal pellets with water and 1% sodium dodecyl sulfate (SDS) was able to improve the purity up to 100% (Murugan et al., 2016b). Later, the 1% SDS was replaced with 0.1 M sodium hydroxide (Ong et al., 2018a). PHA recovered by this method was close to its native morphology in the bacterial cells, while the molecular weights of PHA were not affected (Kunasundari et al., 2017). Currently, this biological recovery method is being scaled-up at a mealworm farm in Malaysia. The entire biological recovery process using mealworms is summarized in Figure 2.

### Other Recovery Methods

Apart from the abovementioned recovery methods, there are several other methods that are still at the laboratory scale. Some of these methods are briefly described and discussed below.

Super critical fluids (SCF) are considered to be a promising future technique to extract PHA from bacterial cells. SCF are solvents with high density and low viscosity. Supercritical-carbon dioxide is the most common SCF solvent for PHA due to its low toxicity, low reactivity, low cost, inflammable temperature and pressure (31°C and 73 atm), and ready availability (Hejazi et al., 2003; Khosravi-Darani et al., 2003; Darani and Mozafari, 2010). Many parameters have to be optimized for this method. However, 89% of PHA has been recovered from *C. necator* using SCF at 200 atm, 40°C, and 0.2 ml of methanol (Hejazi et al., 2003).

Cell fragility is another recovery method, in which the composition of the growth medium is altered to increase the osmotic fragility of the cells after the PHA production phase. Cells grown in inorganic salt media have a deficiency in diaminopimelic acid (DAP) and decreased concentrations of amino acids. DAP is a key component in the bridging of peptidoglycans in the cell wall and hence it plays a crucial role in cell wall stability. About 86–100% of PHA was extracted using hot chloroform. The crucial step in this process is to maintain a balance between cell wall softening when desired and cell wall integrity while PHA is still accumulating (Kunasundari and Sudesh, 2011).

Recovery using gamma radiation provides optimal cell disruption at lower dosage. Following disruption, PHAs from the cells were extracted using organic solvents, alkaline or acidic solutions, surfactants, or enzyme digestion (Bhattacharya, 2000). However, the initial investment cost for this method is extremely high.

### Biodegradation of PHA

In “Agenda 21” adopted at the special United Nations Conference on Environment and Development in 1992, the need for the development and application of new, environmentally friendly processes and materials was emphasized (Sitarz, 1993). Hence, the focus was laid on finding an alternative to petro-plastics due to their non-degradable nature that contributes to the accumulation of solid waste around the world. PHAs hold an exclusive position among biodegradable polyesters with a number of useful properties and a wide range of applications (Cornell, 2007). The attractive properties of PHAs have contributed to its continued studies by both industry and academia.

As the volume of PHA production increases and its application widens, it is important to study the mechanism of biodegradation of PHAs in various environmental conditions. PHAs are degraded by various microorganisms to produce CO_2_ and water in aerobic conditions, and methane and water in anaerobic conditions (Hodzic, 2004; Volova et al., 2010). PHA is able to degrade in most environments that have microbial activities, including soil (Mergaert et al., 1993), compost (Weng et al., 2011), sewage sludge (Lee and Choi, 1999), fresh water, and sea water (Ohura et al., 1999). PHA depolymerases are the key enzymes in the degradation of PHA. The main function of the PHA depolymerase is to hydrolyze the long, water-insoluble PHAs into shorter, water-soluble forms so that microbes can uptake and metabolize the monomers as nutrients (Sudesh and Abe, 2010). The rate of degradation of PHAs is dependent on their characteristics such as monomeric composition, side chain, and crystallinity. The degradation of a particular polymer is also affected by various aspects such as the type of depolymerase, temperature, nutrient availability, and moisture content (Mergaert et al., 1993).

P(3HB) is the most commonly synthesized PHA in most wild-type bacteria. During intracellular degradation, P(3HB) is oxidized by a dehydrogenase and converted into acetyl-CoA by β-ketothiolase and no toxic substances are formed in the cell (Lemes et al., 2015). This contributes to the bio-compatibility of PHAs. Microorganisms capable of producing PHAs express both PHA synthase and a complementary PHA depolymerase.

In general, the degradation rate of PHA will increase with the density of the microbial population. In a study of the degradation of P(3HB-*co*-3HV) by a microbial community, the degrading microbes were found to attach and colonize the polymer before the degrading enzymes were secreted (Sang et al., 2000). Most model microorganisms for PHA research express enzymes with high specificity toward P(3HB). However, various other microbes have also been identified that show a wide substrate specificity (Jendrossek and Handrick, 2002). For instance, genera such as *Xanthomonas* and *Comamonas* are able to degrade PHAs with aromatic side chains (Doi et al., 1992). Later, it was found that clear zones were observed on plates overlaid with P(3HB), P(3HO), and P(3HPV). Hence, Quinteros and co-workers concluded that there is a correlation between the length, composition, and side chains of the polymer and speed of degradation, where longer side-chain PHAs are degraded faster than those with shorter side chains (Quinteros et al., 1999).

Apart from the side chains, composition of the polymer also plays a significant role in degradation. Manna and Paul (2000) evaluated the ability of microbes to degrade P(3HB) and P(3HB-*co*-3HV) in different environments such as soil, water, compost, and sewage sludge. They found that the rate of degradation of homopolymers is higher than for copolymers (Manna and Paul, 2000). These results were in agreement with Doi (1990), who used purified enzymes from *Alcaligenes faecalis* to degrade P(3HB) and P(3HB-*co*-3HV). However, contrasting results were observed in the studies conducted by Mergaert et al. (1992, 1993, 1994), wherein P(3HB-*co*-3HV) degraded at higher rates than P(3HB) in soil and compost environments. Large variations in the rate of degradation are always observed when studies are carried out in natural environments. This is due to variations in conditions observed in different locations as well as the depolymerases present in the resident microorganisms and their substrate specificities (Manna and Paul, 2000).

The PHA degradation ability of microbes is inversely proportional to the crystallinity and the molecular weight of the polymer. This was determined when Kusaka et al. examined the degradation rate of high-molecular-weight 3HB with different crystallinities (65–85%). A similar trend was also observed in another study, which investigated the degradation rate of P(3HB-co-3HV) and P3HB with crystallinity of about 50–65% and 65–80%, respectively. The degradation rate of P(3HB-co-3HV) was 20–30% higher than that of P3HB, when degraded in soil (Kusaka et al., 1999). As the molecular weight of the polymer decreases, the monomers, dimers, and oligomers of the PHA become more available to the microbes (Gu, 2003).

In addition to the abovementioned characteristics of PHAs, the shape also plays a significant role in degradation. For example, thin films of PHA degrade faster than thicker films. A study conducted by Volova et al. (2010) to evaluate the degradation of PHA films and pellets in seawater revealed that the films degraded more compared to the pellets. The higher degradation rate is due to not only the thickness of the PHAs but also the availability of the surface of the PHAs to the microbes (Volova et al., 2010).

The environmental conditions in which PHAs are degraded play a significant role in the rate of degradation. Factors such as soil and climatic conditions influence the PHA degradation rate. A study conducted by Mergaert et al. (1993) stated that the degradation of PHAs are dependent on temperature. They also stated that the degradation of P(3HB) and P(3HB-*co*-3HV) is similar in both sterile buffer as well as in soil at 40°C. Further, Boyandin et al. (2012) evaluated the degradation ability of PHA film in different locations in Vietnam. They concluded that the hot and humid climate of Vietnam facilitated PHA degradation.

## Conclusion

In conclusion, PHA bioplastics have great potential as an alternative to some petro-plastics. Consumer acceptance toward bioplastics over petro-plastic is likely to continue to increase. However, the major restriction for PHAs to enter the market is their higher production cost. Unfortunately, biotechnological production of PHAs has not yet achieved economic viability. However, scientists have made immense progress in searching for a wide array of renewable waste feedstocks for sustainable production of PHAs. In this attractive approach, PHAs are produced sustainably using renewable waste feedstocks, while reducing the volume of waste. Owing to the large amount of waste streams available annually around the globe, waste plant and animal oils are likely to become the primary raw materials for upcoming biotechnological production of PHAs. Undoubtedly, the concept of using waste feedstocks for PHA production is not new, but new and innovative methods for using various untapped waste substrates will continue to be developed and will increase the cost-competitiveness of biotechnologically produced PHAs.

The biosynthesis pattern, structural diversity, and physicochemical properties of the biopolyester have been explored, and the existing research gap in developing this sustainable product has been identified. Efforts have also been made toward the production of PHA using genetically engineered bacterial cells, which are fruitful considering the aspect of the yield and the type of functionalized biopolymer produced. The recent advancements in biosynthesis discussed in this review will lead to greater prospects for PHA market growth. However, research studies of PHA production at the industrial scale to evaluate the efficiency of waste feedstock as a potent carbon source are rather scarce. Overall, sustainable sourcing of feedstock for PHA bioplastic is still in progress, and improvements in the biosynthesis, physiochemical properties, production cost, and biodegradability of PHAs are increasing their competitiveness.

## Author Contributions

AS, ML, JC, AMS, DT, and KS contributed equally for the preparation of this manuscript.

### Conflict of Interest

The authors declare that the research was conducted in the absence of any commercial or financial relationships that could be construed as a potential conflict of interest.
